# Supplementary material for: Specific Circular RNA Signature of Endothelial Cells: Potential Implications in Vascular Pathophysiology
Source: Int J Mol Sci. 2024 Jan 4;25(1):680. doi: 10.3390/ijms25010680 (PMC10779679; doi:10.3390/ijms25010680)

Supplementary Figure S1. RNA sequencing and circRNA identification pipeline

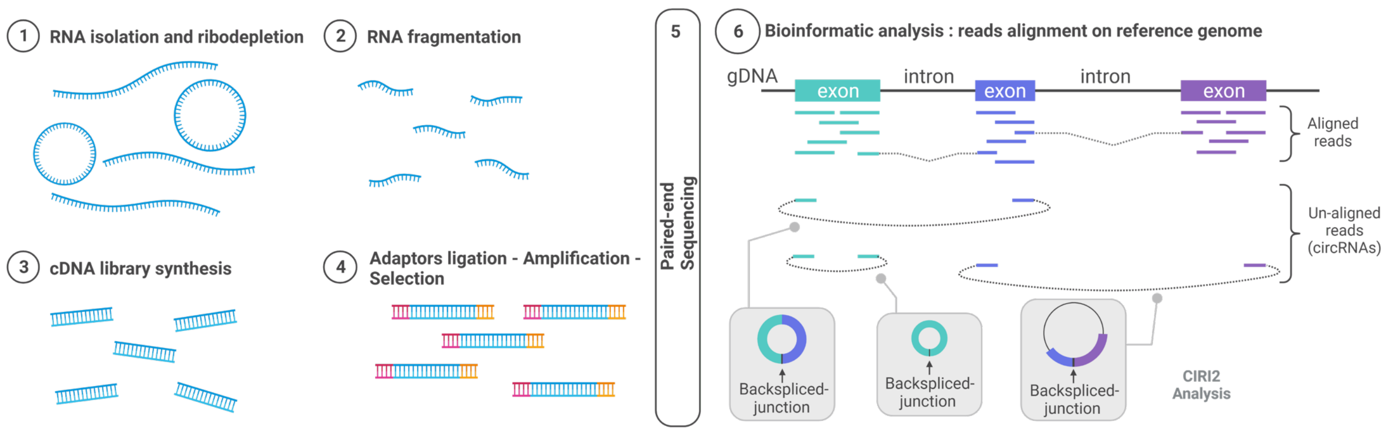

Supplementary Figure S2. CircRNAs genomic localization and transcripts number in all cell lines

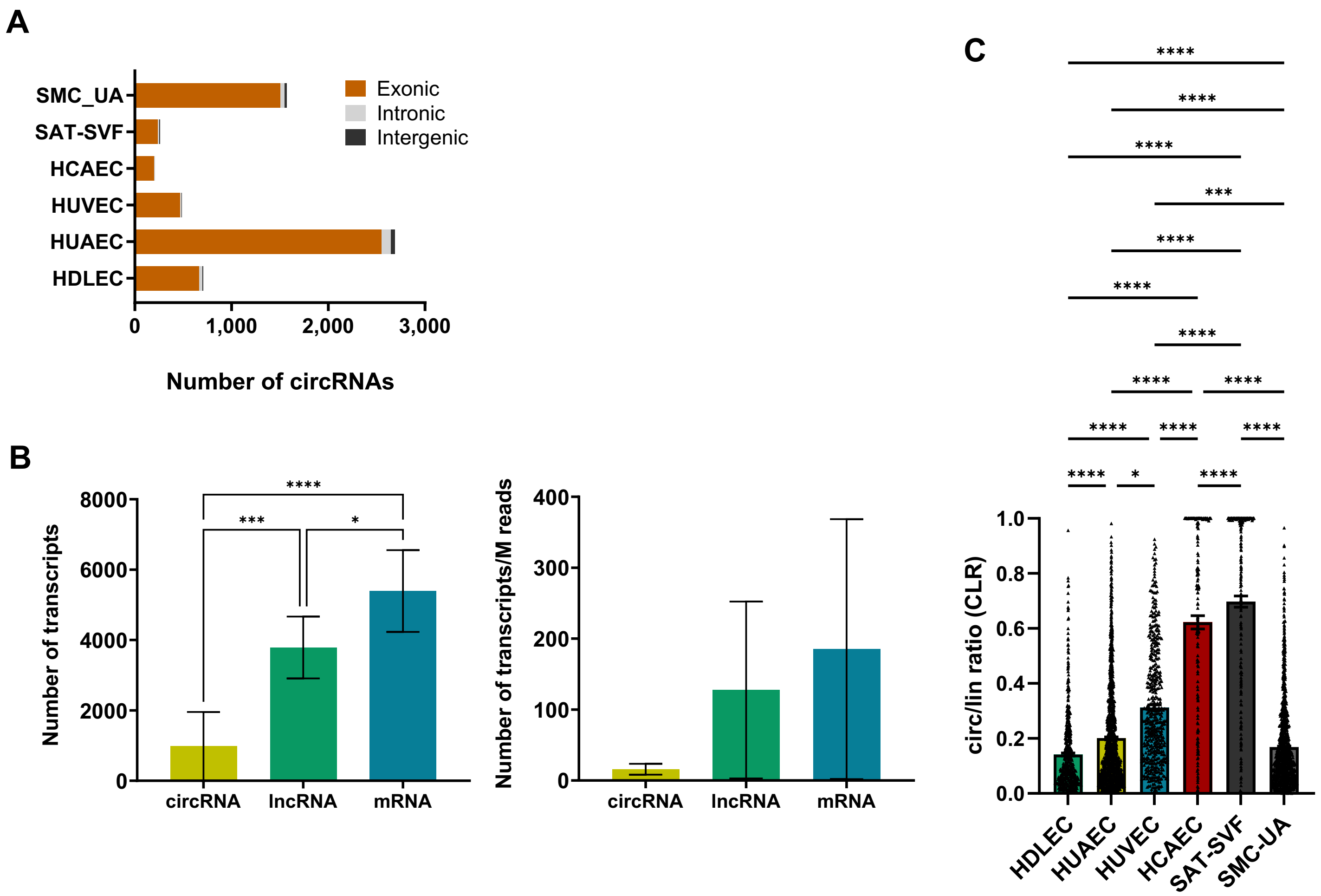

Supplementary Figure S2. CircRNAs genomic localization and transcripts number in all cell lines

(A) Number of distinct circRNAs sorted by genomic origin for each cell type.

(B) Number of distinct transcripts read counts before (left) and after normalization by million (M) of total reads (right) for all cell types combined. Ordinary one-way Anova comparisons p values are plotted on the graphs: *<0,05; ***<0,001; ****<0,0001.

(C) Circular on linear transcript read counts for circRNA expressing genes two-way Anova comparisons: *<0,05; ****<0,0001.

Supplementary Figure S3. Expression of circRNAs as a function of that of their linear counterparts

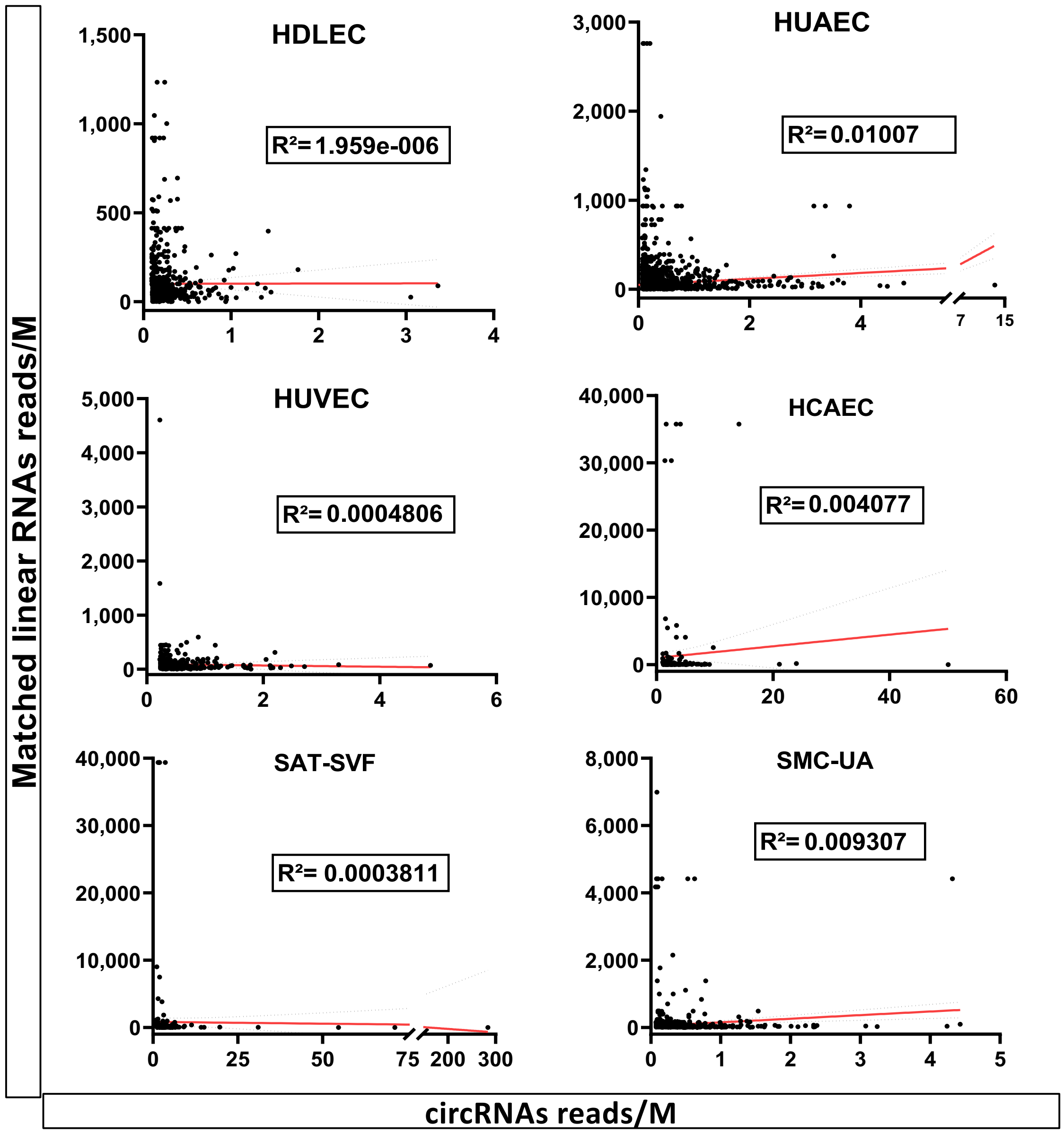

Supplementary Figure S4. Pearson correlation r p values for all transcripts in all cell types

circRNAs Pearson r p values						
	HDLEC	HUAEC	HUVEC	HCAEC	SAT-SVF	SMC-UA
HDLEC		0,0E+00	0,0E+00	1,8E-03	4,3E-01	1,1E-219
HUAEC	0,0E+00		0,0E+00	1,6E-03	7,4E-01	0,0E+00
HUVEC	0,0E+00	0,0E+00		2,8E-04	2,7E-01	0,0E+00
HCAEC	1,8E-03	1,6E-03	2,8E-04		7,9E-64	6,9E-01
SAT-SVF	4,3E-01	7,4E-01	2,7E-01	7,9E-64		1,1E-01
SMC-UA	1,1E-219	0,0E+00	0,0E+00	6,9E-01	1,1E-01	

Matched linear RNAs Pearson r p values						
	HDLEC	HUAEC	HUVEC	HCAEC	SAT-SVF	SMC-UA
HDLEC		4,7E-23	4,5E-44	6,6E-01	5,4E-01	7,5E-01
HUAEC	4,7E-23		2,4E-09	4,5E-01	3,6E-01	2,9E-01
HUVEC	4,5E-44	2,4E-09		1,2E-75	6,6E-148	6,3E-01
HCAEC	6,6E-01	4,5E-01	1,2E-75		2,0E-28	7,2E-01
SAT-SVF	5,4E-01	3,6E-01	6,6E-148	2,0E-28		7,6E-01
SMC-UA	7,5E-01	2,9E-01	6,3E-01	7,2E-01	7,6E-01	

lncRNAs Pearson r p values						
	HDLEC	HUAEC	HUVEC	HCAEC	SAT-SVF	SMC-UA
HDLEC		0,0E+00	0,0E+00	6,2E-08	2,9E-17	0,0E+00
HUAEC	0,0E+00		0,0E+00	1,2E-01	2,8E-01	0,0E+00
HUVEC	0,0E+00	0,0E+00		2,7E-165	1,1E-128	0,0E+00
HCAEC	6,2E-08	1,2E-01	2,7E-165		6,7E-81	1,0E-12
SAT-SVF	2,9E-17	2,8E-01	1,1E-128	6,7E-81		5,3E-06
SMC-UA	0,0E+00	0,0E+00	0,0E+00	1,0E-12	5,3E-06	

mRNAs Pearson r p values						
	HDLEC	HUAEC	HUVEC	HCAEC	SAT-SVF	SMC-UA
HDLEC		0,0E+00	0,0E+00	0,0E+00	0,0E+00	2,1E-179
HUAEC	0,0E+00		0,0E+00	0,0E+00	1,9E-292	0,0E+00
HUVEC	0,0E+00	0,0E+00		0,0E+00	0,0E+00	6,3E-262
HCAEC	0,0E+00	0,0E+00	0,0E+00		0,0E+00	7,7E-187
SAT-SVF	0,0E+00	1,9E-292	0,0E+00	0,0E+00		3,0E-228
SMC-UA	2,1E-179	0,0E+00	6,3E-262	7,7E-187	3,0E-228	

Supplementary Figure S5. Comparison of circRNAs expression across al cell types

A

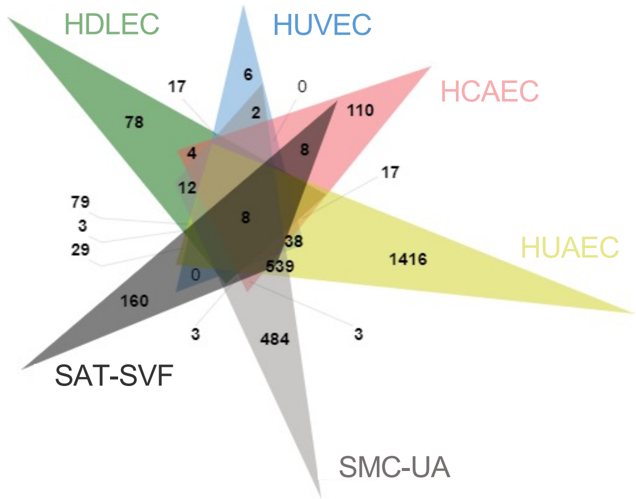

B

Term	Overlap	P-value	Adjusted P-value	Genes
semaphorin-plexin signaling pathway involved in axon guidance (GO:1902287)	1/9	0,0018	0,0252	PLXNA2
motor neuron axon guidance (GO:0008045)	1/10	0,0020	0,0252	PLXNA2
regulation of axon guidance (GO:1902667)	1/13	0,0026	0,0252	PLXNA2
semaphorin-plexin signaling pathway involved in neuron projection guidance (GO:1902285)	1/13	0,0026	0,0252	PLXNA2
cell migration involved in sprouting angiogenesis (GO:0002042)	1/14	0,0028	0,0252	EPHB4
blood vessel endothelial cell migration (GO:0043534)	1/18	0,0036	0,0270	EPHB4
regulation of axon extension involved in axon guidance (GO:0048841)	1/23	0,0046	0,0295	PLXNA2
semaphorin-plexin signaling pathway (GO:0071526)	1/29	0,0058	0,0326	PLXNA2
regulation of axon extension (GO:0030516)	1/34	0,0068	0,0332	PLXNA2
positive regulation of axonogenesis (GO:0050772)	1/37	0,0074	0,0332	PLXNA2

Supplementary Figure S5. Comparison of circRNAs expression across al cell types
(A) Venn diagram representing the comparison of the lists of circRNAs expressed in all ECs.
(B) Enrichr gene ontology analysis terms and p values for the 4 EC-specific circRNAs.

Supplementary Figure S6. Selected specific circRNAs and additional characteristics with sources

circBase_ID	Gene Name	circRNA Name	circRNA localization	Strand	Origin	Genomic lenght (pb)	Spliced lenght (nt)	RBPs (#Binding sites)	miRNA (#Binding sites)	Gene description [sources]	Gene-associated pathologies	Known circRNA functions	References (PMID, doi)
hsa_circ_0111952	PROX1	circPROX1	chr1:213996469-214011715	+	exonic	15247	2095	FMRP (3) IGF2BP2 (4) IGF2BP3 (4)		prospero homeobox 1_protein coding_transcription factor activity-cell specification [GeneCard; HGNC: 9459; NCBI: 5629]	lymphatic malfunctions; cancers; obesity	none	19056879; 16170315; 28024299; 22023334
#N/A	IQSEC1	circIQSEC1	chr3:13129305-13129525	-	intronic	221	221			IQ Motif And Sec7 Domain ArfGEF 1_protein coding_GEF activity-endosomal protein traffic [GeneCards; HGNC: 29112; NCBI: 9922]	intellectual development disorder	none	31607425
hsa_circ_0008285	CDYL	circCDYL	chr6:4891713-4892379	+	exonic	667	667	AGO2 (3) EIF4A3 (4) FMRP (3) HuR (4) IGF2BP3 (4)	hsa-miR-1180 (2)	Chromodomain Y Like_protein coding_histone modification-transcriptional repression [GeneCards; HGNC: 1811; NCBI: 9425]	cancer chemoresistance	pro-myocardial regeneration in vitro	19061646; 31367252; 32522972
hsa_circ_0001727	ZKSCAN1	circZKSCAN1	chr7:100023419-100024307	+	exonic	889	668	EIF4A3 (4) FMRP (5) HuR (7) IGF2BP3 (4)		Zinc Finger With KRAB And SCAN Domains 1_protein coding_transcription factor activity- [GeneCards; HGNC: 13101; NCBI: 7586]	gastric cancer; Hepatocellular cancer	anti-hepatocellular cancers progression	7557990; 28211215; 33439397
hsa_circ_0005895	CARD6	circCARD6	chr5:40852174-40854096	+	exonic	1923	1923	AGO2 (5) EIF4A3 (6)	hsa-miR-599 (3)	Caspase Recruitment Domain Family Member 6_protein coding [GeneCards; HGNC: 16394; NCBI: 84674]	inflamatory bowel diseases	pro-posterior capsul opacification (eye)	12775719; 16418290; 20025480; 33844960;
hsa_circ_0002472	PLXNA2	circPLXNA2	chr1:208210280-208218002	-	exonic	7723	1451	AGO2 (3) EIF4A3 (10) FUS (3) HuR (3)	hsa-miR-1205 (3) hsa-miR-1286 (3)	Plexin A2_protein coding [GeneCards; HGNC: 9100; NCBI: 5362]	axonal guidance disfunctions; Breast cancer tumorigenesis	pro-proliferation and anti-apoptosis in myoblasts	16402134; 21925246; 10.3390/ijms24065459
hsa_circ_0000914	FKBP8	circFKBP8	chr19:18537601-18538436	-	exonic	836	394	EIF4A3 (3) FMRP (3)		FKBP Prolyl Isomerase 8_protein coding [GeneCards; HGNC: 3724; NCBI: 23770]	spina bifida	none	18003640; 32969478
#N/A	ERCC6L2	circERCC6L2	chr9:95978061-96004701	+	exonic	26641	337			ERCC Excision Repair 6 Like 2_protein coding [GeneCards; HGNC: 26922; NCBI: 375748]	bone marrow failure syndrome	none	4507776
#N/A	FIRRE	circFIRRE	chrX:131749306-131794466	-	exonic	45161	872			Firre Intergenic Repeating RNA Element_lncRNA [GeneCards; HGNC: 49627; NCBI: 286467]	various cancers	chrX inactivation and nuclear organisation? RNA stability?	35110535; 35988459; 29678151; 30124921;
hsa_circ_0007026	ZMYND8	circZMYND8_A	chr20:47262288-47276795	-	exonic	14508	623	AGO2 (4) EIF4A3 (3)	hsa-miR-370 (2) hsa-miR-625 (2)	Zinc Finger MYND-Type Containing 8_protein coding [GeneCards; HGNC: 9397; NCBI: 23613]	various cancers	none	11003709; 27477906
hsa_circ_0003028	FUT8	circFUT8	chr14:65561337-65561766	+	exonic	430	430	AGO2 (2) HuR (4)	hsa-miR-1305 (2)	Fucosyltransferase 8_protein coding [GeneCards; HGNC: 4019 NCBI: 2530]	various cancers; congenital disorders	cancer suppression or promotion?	19302290; 26289314; 29304374; 32072011; 33500381
#N/A	SERPINE1	circSERPINE1	chr7:101131916-101135801	+	exonic	3886	541			Serpin Family E Member 1_protein coding [GeneCards; HGNC: 8583 NCBI: 5054]	Thrombosis; PAI1-deficiency	none	3922531; 9207454
hsa_circ_0087960	LPAR1	circLPAR1	chr9:110972073-110973558	-	exonic	1486	226	None		Lysophosphatidic Acid Receptor 1_protein coding [GeneCards; HGNC: 3166 NCBI: 1902]	pertusis	invasive bladder cancer biomarker	30867795; 9804623
#N/A	ABHD14B	circABHD14B	chr3:51968816-51969034	+	exonic	219	219			Abhydrolase Domain Containing 14B_protein coding [GeneCards; HGNC: 28235 NCBI: 84836]	none	none	
hsa_circ_0128684	HNRNPAB	circHNRNPAB	chr5:178210635-178210877	+	exonic	243	243	AGO2 (4) FMRP (4) HuR (3) LIN28A (3)	hsa-miR-625 (2)	Heterogeneous Nuclear Ribonucleoprotein A/B_protein coding [GeneCards; HGNC: 5034 NCBI: 3182]	none	none	
hsa_circ_0005015	HAS2	circHAS2	chr8:121628714-121629340	-	exonic	627	627	EIF4A3 (6)	hsa-miR-519d-3p	Hyaluronan Synthase 2_protein coding [GeneCrads; HGNC: 4819 NCBI: 3037]	breast cancer	diabeties retinopathy biomarker	29288268; 22113945; 33954907
hsa_circ_0001461	FAT1	circFAT1	chr4:186706563-186709845	-	exonic	3283	3283	AGO2 (19) EIF4A3 (26) FMRP (25) HuR (17) IGF2BP1 (13) IGF2BP2 (17) IGF2BP3 (18) LIN28A (15) PTB (15)	hsa-miR-1183 (3) hsa-miR-1208 (5) hsa-miR-1229 (2) hsa-miR-1231 (2) hsa-miR-1236 (3) hsa-miR-1238 (2) hsa-miR-1245 (2) hsa-miR-1248 (2) hsa-miR-1287 (3) hsa-miR-1305 (2) hsa-miR-142-3p (2) hsa-miR-145 (2) hsa-miR-186 (2) hsa-miR-198 (2) hsa-miR-326 (2) hsa-miR-330-5p (2) hsa-miR-338-3p (2) hsa-miR-377 (2) hsa-miR-507 (2) hsa-miR-515-5p (2) hsa-miR-520f (2) hsa-miR-548g (5) hsa-miR-548p (2) hsa-miR-557 (2) hsa-miR-570 (2) hsa-miR-579 (2) hsa-miR-580 (2) hsa-miR-587 (3) hsa-miR-616 (2) hsa-miR-626 (2) hsa-miR-662 (2) hsa-miR-668 (2) hsa-miR-7 (2) hsa-miR-4781-3p	FAT Atypical Cadherin 1_protein coding [GeneCards; HGNC: 3595 NCBI: 2195]	various cancers	pro-cancer cell stemness; pro-breast cancer drug resistance; osteoblast differenciation	34314629; 34288822; 35003269; 23354438;

Supplementary Figure S7. Schematic representation of selected circRNAs and their structures

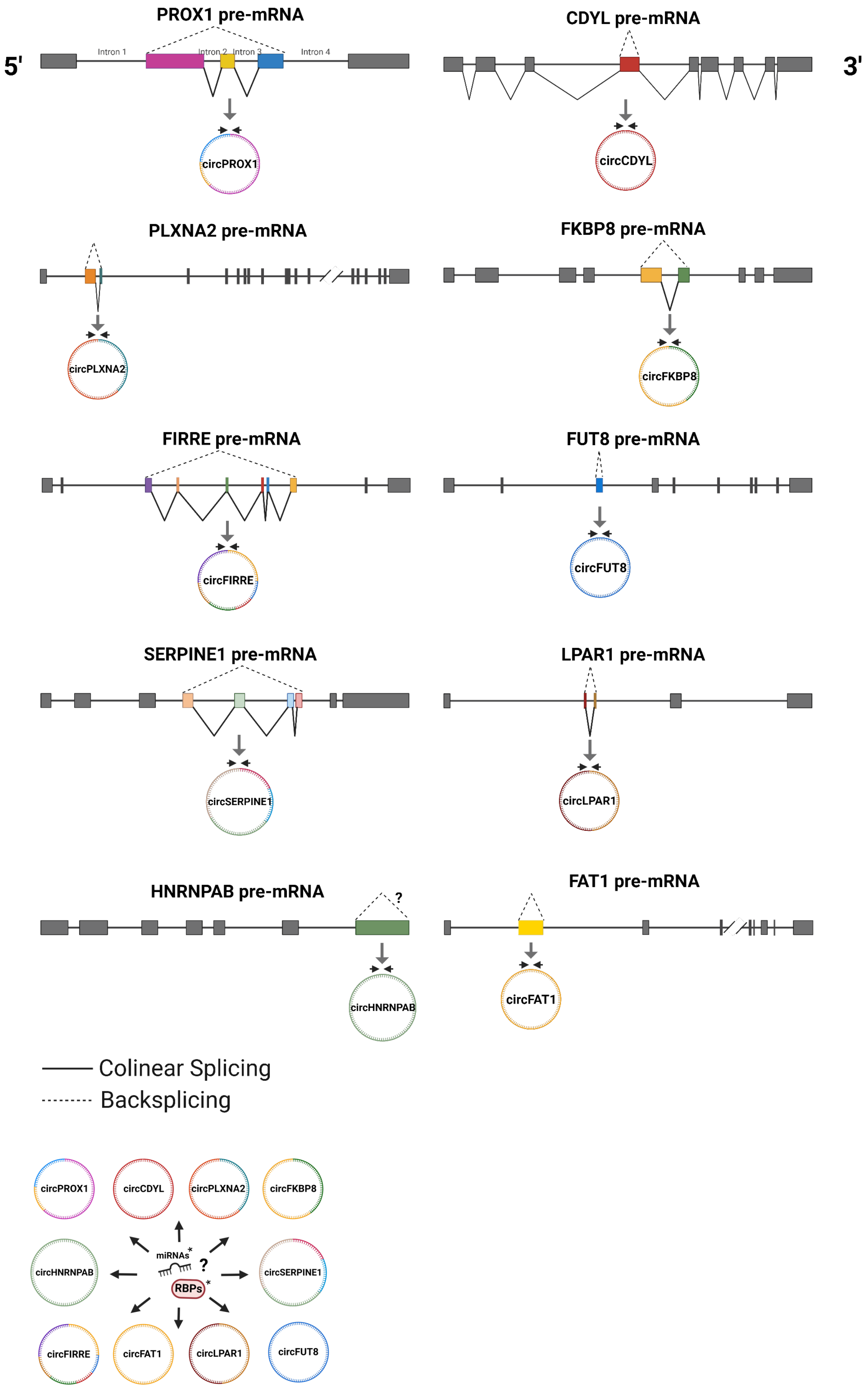

Supplementary Figure S8. RNAseq expression levels for selected transcripts in all cell types

A

circRNAs (reads/Million)						
Name	HDLEC	HUAEC	HUVEC	HCAEC	SAT-SVF	SMC-UA
circPROX1	1,42	0,00	0,00	0,00	0,00	0,00
circIQSEC1	0,94	0,00	0,00	0,00	0,00	0,00
circCDYL	0,89	3,12	2,29	6,06	0,38	21,92
circZKSCAN1	0,83	2,94	2,48	1,85	0,92	27,98
circCARD6	0,42	0,77	0,39	1,79	0,00	0,00
circPLXNA2	0,25	0,51	0,60	2,58	0,00	0,00
circFKBP8	0,00	1,52	0,32	0,00	0,00	4,09
circERCC6L2	0,00	1,36	0,32	0,00	0,00	5,81
circFIRRE	0,00	0,93	0,00	0,00	0,00	0,00
circZMYND8_A	0,00	0,00	0,40	0,00	0,00	0,00
circFUT8	0,00	0,00	0,28	0,00	0,00	0,00
circSERPINE1	0,00	0,00	0,00	14,16	0,00	0,00
circLPAR1	0,00	0,00	0,00	0,00	0,60	4,14
circABHD14B	0,00	0,00	0,00	0,00	0,45	0,00
circHNRNPAB	0,00	0,00	0,00	0,00	0,43	0,00
circHAS2	0,00	0,00	0,00	0,00	0,00	34,09
circFAT1	0,00	0,00	0,00	0,00	0,00	19,39

matched linRNAs (reads/Million)						
Name	HDLEC	HUAEC	HUVEC	HCAEC	SAT-SVF	SMC-UA
PROX1	396,63	0,35	3,52	8,86	0,00	1,36
IQSEC	59,019	8,619	8,212	17,228	2,574	148,131
CDYL	20,60	16,09	29,39	26,06	1,21	87,37
ZKSCAN1	79,25	57,57	61,22	50,11	2,42	490,56
CARD6	60,80	21,85	39,02	18,32	0,83	17,63
PLXNA2	177,00	170,50	168,54	920,79	1,52	54,44
FKBP8	67,71	58,22	122,53	97,01	10,72	551,57
ERCC6L2	19,07	29,46	13,99	5,87	0,63	199,70
FIRRE	0,50	11,27	2,28	0,00	0,00	3,13
ZMYND8	74,89	22,55	77,01	38,29	2,26	164,29
FUT8	5,83	22,02	21,13	16,60	0,68	245,05
SERPINE1	87,38	1400,27	2488,48	35759,38	50,91	19085,71
LPAR1	0,00	0,75	0,00	1,63	13,28	266,92
ABHD14B	10,49	2,26	15,59	177,96	16,17	28,94
HNRNPAB	98,14	35,91	112,44	38,18	2,27	110,61
HAS2	0,00	0,91	0,38	0,00	0,23	816,36
FAT1	0,81	23,92	18,31	4,89	16,59	4882,22

B

circ/lin ratio (CLR)						
Name	HDLEC	HUAEC	HUVEC	HCAEC	SAT-SVF	SMC-UA
circPROX1	0,17	#N/A	#N/A	#N/A	#N/A	#N/A
circIQSEC1	0,77	#N/A	#N/A	#N/A	#N/A	#N/A
circCDYL	0,53	0,81	0,76	0,71	0,65	0,74
circZKSCAN1	0,27	0,66	0,66	0,50	0,82	0,49
circCARD6	0,15	0,49	0,30	0,64	#N/A	#N/A
circPLXNA2	0,09	0,11	0,22	0,35	#N/A	#N/A
circFKBP8	#N/A	0,21	0,03	#N/A	#N/A	0,05
circERCC6L2	#N/A	0,69	0,55	#N/A	#N/A	0,38
circFIRRE	#N/A	0,72	#N/A	#N/A	#N/A	#N/A
circZMYND8_A	#N/A	#N/A	0,19	#N/A	#N/A	#N/A
circFUT8	#N/A	#N/A	0,23	#N/A	#N/A	#N/A
circSERPINE1	#N/A	#N/A	#N/A	0,00	#N/A	#N/A
circLPAR1	#N/A	#N/A	#N/A	#N/A	0,47	0,15
circABHD14B	#N/A	#N/A	#N/A	#N/A	0,10	#N/A
circHNRNPAB	#N/A	#N/A	#N/A	#N/A	0,41	#N/A
circHAS2	#N/A	#N/A	#N/A	#N/A	#N/A	0,38
circFAT1	#N/A	#N/A	#N/A	#N/A	#N/A	0,22

Supplementary Figure S9. qPCR relative expression levels for selected transcripts in all cell types

circRNAs (RT-qPCR)							matched linRNAs (RT-qPCR)							Coherence
Name	HDLEC	HUAEC	HUVEC	HCAEC	SAT-SVF	SMC-UA	Name	HDLEC	HUAEC	HUVEC	HCAEC	SAT-SVF	SMC-UA	
circPROX1	1,00	0,00	0,00	0,00	0,00	0,00	PROX1	1,00	0,00	0,00	0,00	0,02	0,00	YES
circIQSEC1	1,00	0,00	0,00	0,00	0,02	0,00	IQSEC	1,00	0,02	0,02	0,02	0,25	0,02	NO
circCDYL	1,00	2,21	0,71	1,44	2,91	1,03	CDYL	1,00	0,52	0,36	0,27	0,98	0,22	NO
circZKSCAN1	1,00	2,04	0,64	0,83	2,42	0,56	ZKSCAN1	1,00	0,26	0,28	0,18	0,69	0,10	NO
circCARD6	1,00	0,15	0,22	0,29	3,65	0,21	CARD6	1,00	0,60	0,39	0,21	1,64	0,12	NO
circPLXNA2	1,00	0,64	0,98	1,00	0,05	0,17	PLXNA2	1,00	0,38	0,34	0,54	0,14	0,02	NO
circFKBP8	1,00	1,41	0,43	0,98	5,45	0,65	FKBP8	1,00	0,30	0,40	0,32	1,47	0,43	NO
circERCC6L2	1,00	0,94	0,34	0,29	1,64	0,73	ERCC6L2	1,00	0,28	0,33	0,06	1,10	0,05	NO
circFIRRE	1,00	8,85	1,48	1,38	1,25	0,21	FIRRE	1,00	10,30	0,10	0,00	0,02	0,08	YES
circZMYND8_A	1,00	0,76	0,43	0,33	0,60	0,19	ZMYND8	1,00	0,12	0,21	0,05	0,26	0,04	NO
circFUT8	1,00	7,81	5,96	13,34	6,63	10,82	FUT8	1,00	1,97	3,26	3,87	6,15	2,14	NO
circSERPINE1	1,00	1,50	0,71	1355,31	13,52	0,47	SERPINE1	1,00	1,74	1,23	1,05	0,19	1,50	NO
circLPAR1	1,00	4401,30	3622,10	3,38	1555217,12	74697,84	LPAR1	1,00	2075,32	5392,33	164,12	896301,20	84534,65	NO
circABHD14B							ABHD14B	1,00	0,13	0,16	0,20	1,53	0,15	NO
circHNRNPAB	1,00	0,91	1,03	0,82	5,09	1,00	HNRNPAB	1,00	1,01	0,86	1,13	1,96	0,40	NO
circHAS2	1,00	556,76	189,88	66,90	8207,32	15330,11	HAS2	1,00	105,36	41,03	2,01	6952,36	995,22	NO
circFAT1	1,00	1,31	3,18	0,01	0,04	143,98	FAT1	1,00	3,29	1,51	0,04	50,04	29,04	NO

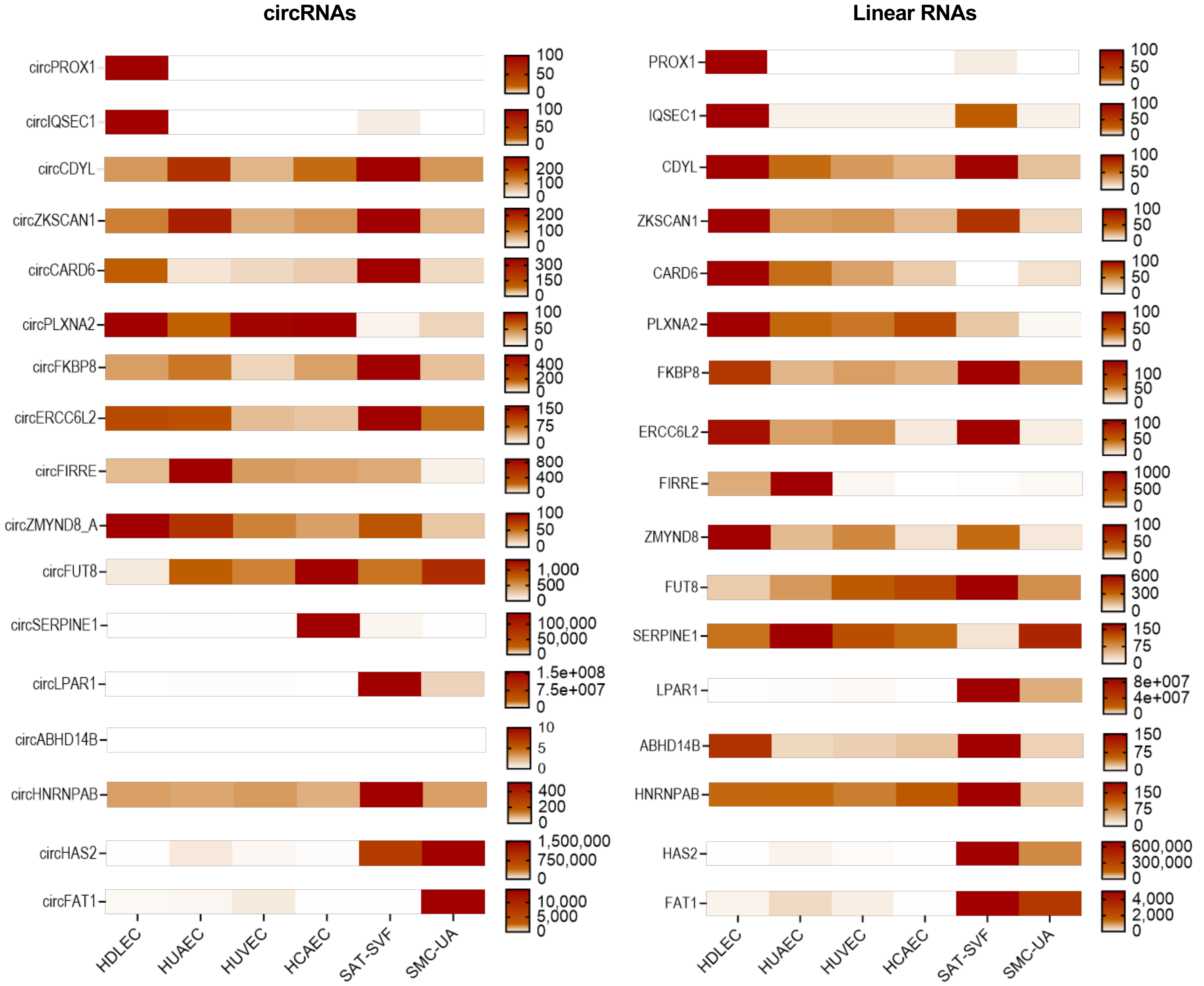

Supplement: Supplementary file 1 [file ijms-25-00680-s001.zip › ijms-2758981-supplementary.pdf]
